# Impacts of active urea secretion into pars recta on urine concentration and urea excretion rate

**DOI:** 10.1002/phy2.34

**Published:** 2013-08-22

**Authors:** Anita T Layton, Lise Bankir

**Affiliations:** 1 Department of Mathematics, Duke UniversityDurham, 27708-0320, North Carolina; 2 INSERM, Unité 872–E2, Centre de Recherche des CordeliersParis, 75006, France

**Keywords:** Collecting duct, loop of Henle, thin descending limbs, urea transporter, UT-A2

## Abstract

It has been observed experimentally that early distal tubular urea flow exceeds urea delivery by the proximal convoluted tubule to the pars recta and loop of Henle. Moreover, the fractional excretion of urea in the urine may exceed values compatible with the reabsorption known to occur in the proximal convoluted tubule in the cortex. A likely explanation for these observations is that urea may be actively secreted into the pars recta, as proposed in a few studies. However, this hypothesis has yet to be demonstrated experimentally. In this study, we used a mathematical model of the renal medulla of the rat kidney to investigate the impacts of active urea secretion in the intrarenal handling of urea and in the urine concentrating ability. The model represents only the outer and inner medullary zones, with the actions taking place in the cortex incorporated via boundary conditions. Blood flow in the model vasculature is divided into plasma and red blood cell compartments. We compared urea flow rates and other related model variables without and with the hypothetical active urea secretion in the pars recta. The simulation suggests that active urea secretion induces a “urea-selective” improvement in urine concentrating ability by enhancing the efficiency of urea excretion without requiring a higher urine flow rate, and with only modest changes in the excretion of other solutes. These results should encourage experimental studies in order to assess the existence of an active urea secretion in the rodent kidney.

## Introduction

Most mammals are able to produce a urine that is more concentrated than blood plasma, a process that is permitted by the anatomical and functional adaptations of the kidney, not found in lower vertebrates. In omnivores and carnivores, urea is the most abundant solute in the urine but its concentration in the blood plasma is relatively low (4–10 mmol/L), compared to other solutes such as sodium (140 mmol/L). Urea, which represents only about 2% of the solutes filtered by the glomeruli, makes up about 40–50% of all solutes in the urine (or even more depending on the protein content of the diet). Accordingly, urea is concentrated up to 100 times in urine with respect to plasma in humans, and up to 1000 times in mice and some desert-adapted rodents (Bankir and de Rouffignac 1985). Concentrating a solution requires energy. The active sodium reabsorption that occurs in the thick ascending limbs and collecting ducts allows the accumulation of concentrated sodium chloride in the medulla. The concentration of some other solutes like potassium, ammonium or protons is achieved by an active (energy-dependent) secretion occurring in specific segments of the nephron equipped with specialized membrane transporters. However, the mechanisms that allow the concentration of urea to the level observed in the mammalian kidney (and especially in rodents) are still unclear. An active or secondary active secretion of urea is usually not considered in our present concepts of the urine-concentrating mechanism.

The current view, summarized in many reviews and chapters (Knepper and Roch-Ramel 1987; Bankir and Trinh-Trang-Tan 2000; Yang and Bankir 2005; Sands and Layton 2009), states that a vasopressin-dependent increase in urea permeability of the terminal inner medullary collecting duct (IMCD) (via facilitated urea transporters UT-A1/3/4) delivers significant amounts of filtered urea, concentrated upstream by water reabsorption, in the deep inner medulla (IM). Urea is then “sequestrated” in the medulla (Valtin 1966) by a complex intrarenal “recycling” via both a vascular and a tubular pathway involving the urea transporters UT-B and UT-A2, respectively. While the contribution of countercurrent exchange between ascending and descending vasa recta has been confirmed by the defects observed in UT-B knockout mice (Yang et al. 2002; Bankir et al. 2004), some doubts have emerged regarding the role of UT-A2 in this recycling, in part because UT-A2 knockout mice do not exhibit the expected urine concentrating defect (Uchida et al. 2005).

Micropuncture experiments have shown that about 50% of filtered urea is reabsorbed in the proximal convoluted tubule accessible to micropuncture and that this fraction is not altered by the hydration state of the animal (Armsen and Reinhardt 1971). Accordingly, about 50% of the filtered urea is delivered to the loops of Henle. However, urea flow rate in the early distal tubule of superficial nephrons (accessible to micropuncture at the kidney surface) substantially exceeds 50% of the filtered urea in rat and in several other rodents (Lassiter et al. 1961; De Rouffignac and Morel 1969; Armsen and Reinhardt 1971; de Rouffignac et al. 1981; Bankir and Trinh-Trang-Tan 2000), indicating that some urea must be added into the descending branch of the loops of Henle (the flow of urea at the tip of Henle's loops of long-looped nephrons also exceeds 50% of the estimated filtered load of urea). Moreover, the fractional excretion of urea has often been reported to exceed 50%, and even in a few cases 100% (Bankir and Trinh-Trang-Tan 2000). In general, a threshold of 100% is required to assume the existence of an active secretion of an arbitrary solute. In the case of urea, the fraction reabsorbed in the proximal convoluted tubule (about 50% as indicated above) is driven by an intense cortical blood flow back to the general circulation, and thus cannot reenter the nephron. Accordingly, any figure of fractional excretion of urea above 50% (not 100% as often assumed) suggests that some net tubular secretion occurs (Bankir and Trinh-Trang-Tan 2000; Yang and Bankir 2005; Bankir and Yang 2012). However, the source of the secreted urea has yet to be determined.

Bankir and Yang (2012) recently reviewed a number of observations, in different species including humans, that suggest the existence of active urea secretion that probably takes place in the S3 segment of the straight proximal tubule in the deep cortex and the outer stripe of the outer medulla (OM). By extracting urea from the medullary vasculature, this active secretion delivers urea in the nephron lumen in addition to the previously filtered urea that is delivered to the IM through the terminal collecting duct and recycled by countercurrent exchange. This active secretion may account for the difference between the urea flow remaining in the late superficial proximal convoluted tubule and the urea flow observed in the early distal tubule, as well as for fractional excretion of urea exceeding 50% (Bankir and Yang 2012).

In this study, we have added active urea secretion to a previous mathematical model of the urine concentrating mechanism of the medulla of the rat kidney (Layton et al. 2012). The resulting model was used to study the potential impacts of the hypothesized active urea secretion into pars recta on urine concentration and urinary urea and other solute excretion.

## Model Formulation

Our mathematical model is an extension of the “region-based” model of the renal medulla of the rat kidney (Layton 2011). The model includes loops of Henle, vasa recta, red blood cells (RBC), and the collecting duct (CD) system, which are represented by rigid tubes that extend along the cortico-medullary axis. Two thirds of the model loops of Henle turn at the outer–inner medullary border, and the remainder turn at all levels of the IM. The model represents vasa recta that terminate or originate at all levels of the medulla, as well as a composite CD.

Anatomical studies have revealed that in the OM of the rat kidney, tubules are organized around vascular bundles (Kriz 1967; Kriz et al. 1972). That radial organization is represented in the model by means of four interconnected regions (Layton and Layton 2005; Layton 2011). The portion of each region that is exterior to both tubules and vasa recta represents merged capillaries, interstitial cells, and interstitial space. Detailed anatomical studies in rats and mice have also shown that in the upper 3.5 mm of the IM, clusters of IMCD provide the organizing motif around which loops of Henle and vessels are arranged (Pannabecker and Dantzler 2004, 2007). That radial organization is represented in the model by means of three interconnected regions (Layton 2011).

The model is formulated for five solutes: NaCl, urea, a non reabsorbable solute (loosely associated with K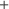), proteins, and hemoglobin. NaCl is represented by Na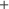. NaCl and urea are assumed to be present in the tubular fluid, vascular fluid, interstitial fluid, and in the RBCs. In the RBCs, which are impermeable to Na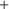, Na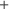 also represents other non urea solutes. The non reabsorbable solute, denoted “NR,” is assumed to be present in significant amounts only in the tubular fluid of the CD; therefore, in the model, NR is represented only in CD tubular fluid. The glomerular filtrate is assumed to be mostly free of large proteins; thus, proteins are represented only in the plasma fluid and in the interstitium. Hemoglobin is represented only in the RBCs.

The model predicts fluid flow, solute concentrations, transmural water and solute fluxes, and fluid osmolality, as functions of medullary depth, in the tubules, vessels, interstitium, and RBCs. The model equations, which can be found in Layton and Layton (2005), Layton (2007), Layton et al. (2010) and Layton (2011), are based on the principle of mass conservation of both solutes and water, and on single-barrier transmural transport equations that approximate double-barrier transepithelial and transendothelial transport processes. Transmural solute diffusion for loops of Henle and CDs is characterized by solute permeabilities. Active transport is approximated by a saturable expression having the form of Michaelis-Menten kinetics; the transport equations for water represent osmotically driven fluxes (except for AVR which are fenestrated and thus freely permeable to water and solutes).

The transmural flux of solute *k* from region *m* into a loop of Henle or CD (denoted 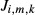, where *i* denotes loop or CD) is given by


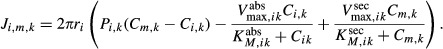
(1)

The first term inside the parentheses on the right is transmural diffusion characterized by permeability 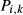. The second and third terms represent outward- and inward-directed active transport by a saturable expression having the form of Michaelis-Menten kinetics. They are characterized by Michaelis constants 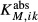 and 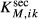 and maximum transport rates 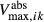 and 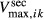, respectively. Active NaCl reabsorption is known to be significant only along the thick ascending limbs and, to a much smaller extent, some descending limb segments. Thus, 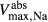 is non zero only along these segments, and 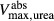 is assumed to be zero. Active urea secretion is assumed to take place along the pars recta only. NaCl is not assumed to be actively secreted.

The transport parameters are shown in Table 1; these parameters are discussed in Layton and Layton (2005), Chen et al. (2009), Layton et al. (2010) and Layton (2011). The maximum active transport rates are shown in Table 1. Active urea transport was assumed to be present only along the pars recta and to be inward-directed. The Michaelis constant for Na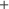 active transport 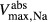 was set to 45 mmol/L (Greger and Velázquez 1987), and for urea active transport 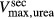 was set to 15 mmol/L. These parameters were chosen to produce a urea secretion rate that is approximately 50% of the urea filtration rate (summed over all nephrons, including short ones), as suggested in Safirstein et al. (1981) and Bankir and Yang (2012).

**Table 1:** Tubular diameters and transport parameters

Tubule or vessel	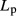 (cm mmHg/sec)	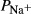 ( 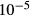 cm/sec)	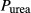 ( 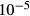 cm/sec)	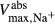 (nmole/[cm 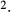 sec])	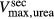 (nmole/[cm 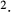 sec])
PST	3.36 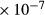	10	1.5	2.1	10
SDL	3.06 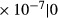	1.5 | 1.1	7.4 | 200	0.43 | 0	0
OM LDL	2.16 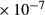	63	0.5	0.43	0
IM LDL	2.07 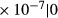	0	13 | 180	0	0
IM LDL 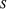	0	0	200	0	0
SAL	0	1.1	1.4 | 0.9	10.5 | 25.9	0
OM LAL	0	1.1	1.4 | 0.6	10.5 | 25.9	0
IM LAL	0	80	190	0	0
OMCD	4.23 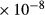	1	0.3	0	0
IMCD	4.23 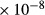	1	0.5 to 110	8.5 → 4	0

PST, proximal straight tubule; SDL/LDL, short/long descending limb; SAL/LAL, short/long ascending limb; CD, collecting duct; LDL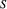, LDL that turns within the first mm of the IM. Arrow (→) indicates that parameter is assumed to vary linearly as *x* increases; vertical line (|) indicates that parameter is assumed to change abruptly. Axial variations in IMCD 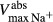 are given in Ref. (Layton et al. (2012)).

The boundary concentrations and water flows for descending limbs and DVR at the cortico-medullary border (*x* = 0) are given in Table 2. The assumptions on which the boundary conditions for CD inflow were based can be found in Layton and Layton (2011). Those assumptions suffice to determine the CD fluid inflow rate, Na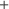, and urea concentrations (Layton and Layton 2003, 2005). In this study, we assume that 35% of the urea that is delivered to the early distal tubule by the cortical ascending limb is absorbed in the cortex (increased from 20% in Layton 2011 to yield a realistic OMCD urea flow rate, given the significantly higher urea flow rate along the thick ascending limbs when active urea secretion is represented). And we assume that 84% of the fluid delivered to the early distal tubule is reabsorbed along the distal tubule and cortical CD.

**Table 2:** Boundary conditions at the cortico-medullary border

Structure	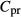 (mmol/L)	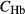 (mmol/L)	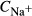 (mmol/L)	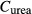 (mmol/L)	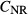 (mmol/L)	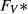 (nL/min)	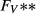 (nL/min)
SDL	0	0	162	11	0	10	6.67
LDL	0	0	162	11	0	12	4
DVR	6.8	0	164	8	0	6‡	14.7‡
RBC	0	5.1	164§	8	0	2	4.91
CD	0	0	78.4	157	7	6.91	1.13

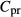
 and 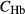, concentrations of plasma proteins and hemoglombin. Flow rates 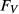 are given for two scalings: per individual tubule or vessel (*) and per nephron (**).

‡ Plasma flow only, based on 0.25 hematocrit.

§ Includes both Na^+^ and non reabsorbable solutes.

In the OM, long AVR are assumed to be distributed within the vascular bundles. Two long AVR are represented in the OM: one, denoted “LAV1,” is assumed to occupy the central part of the vascular bundles, and the other, denoted “LAV2,” is found within the periphery of the vascular bundles. The model assumes that 90% of the AVR arising from the IM region that contains the DVR (these AVR are denoted “LAV6”) enter the OM as LAV1, and the remainder of the AVR (labeled “LAV5” and “LAV7”) join LAV2. Thus, at the OM-IM border we impose the following conditions


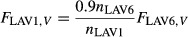
(2)


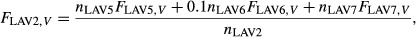
(3)


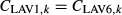
(4)


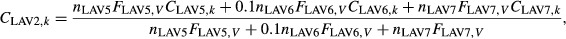
(5)

where 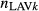 denotes the proportion of LAV*k* (*k* = 1,…,7) per nephron.

## Results

**Figure 1:**
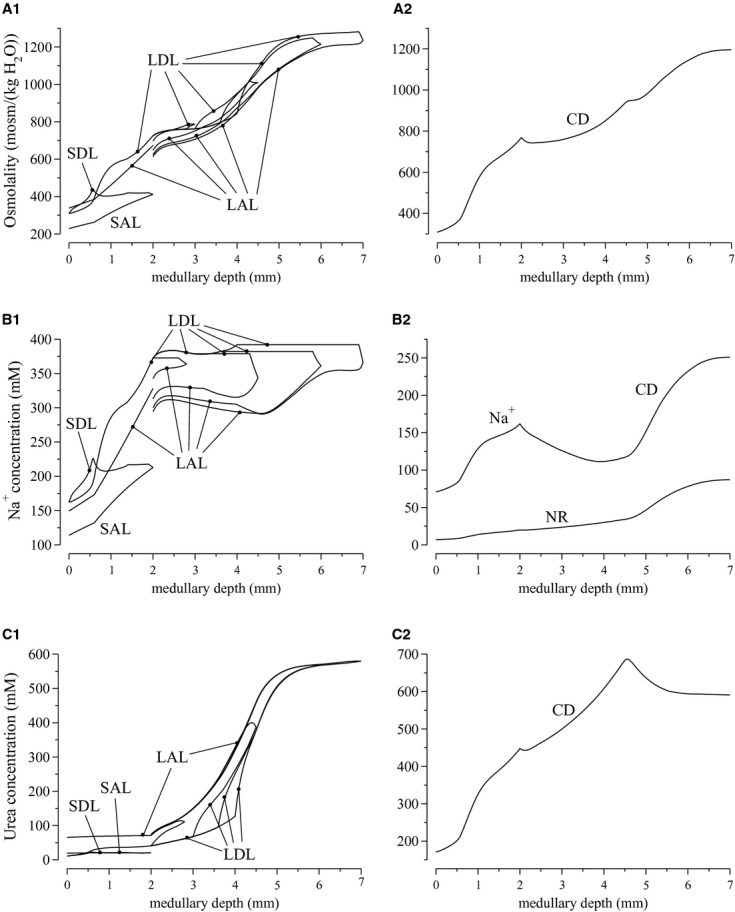
Osmolalities and concentration profiles of loops of Henle (left panels) and collecting duct (CD) (right panels), for the “With urea secretion” case. SDL/LDL, short/long descending limb; SAL/LAL, short/long ascending limb. The ordinate is identified at the top of each column: A, osmolality; B, Na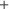 or NR concentration; C, urea concentration. Lengths of outer stripe, inner stripe, and IM are 0.6, 1.4, and 5 mm. Note variation, among panels, in ordinate scalings.

**Figure 2:**
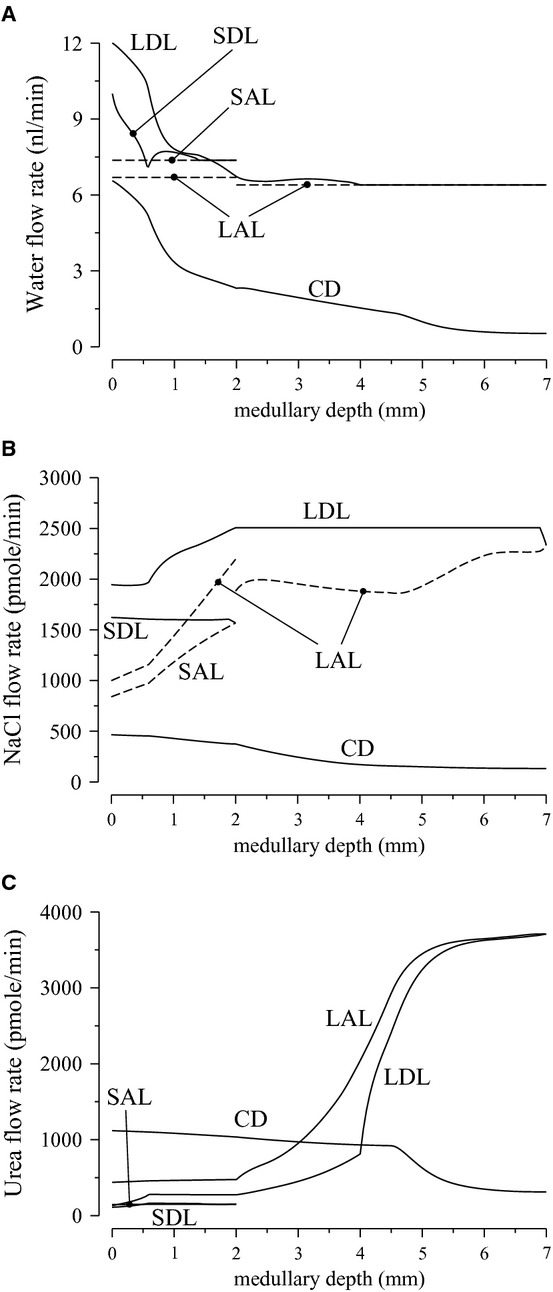
Water and solute flows of loops and Henle and collecting duct (CD) for the “With urea secretion” case. A, water flow; B, Na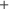 flow; C, urea flow. Notations are analogous to Figure 1. Flows toward the cortex and the papillary tip are denoted by dashed lines and solid lines, respectively. The OM segments of the long-loop profiles show average values of all long loops; the IM segments correspond to the loop that reaches to the papillary tip.

To assess the impacts of urea secretion on the urine concentrating mechanism, we computed steady-state model solutions for two cases: one with active urea secretion into pars recta, and the other without (base case). In the base case, active urea secretion was prevented by setting 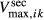 to 0. Key results for the “With urea secretion” case are displayed graphically in Figures 1 and 2. In the model, the loops of Henle are represented by a continuous distribution, with the loops turning at all IM levels. However, Figure 1 contains only the profiles that correspond to representative long loops of Henle, specifically, those that turn at *x* = 3.0, 4.5, 6.0, and 7.0 mm. The OM segments of the long-loop profiles in Figure 2 depict aggregate flows of all long loops; the IM segments correspond to the longest loop.

A comparison of urine composition and excretion rates obtained for the two cases is shown in Table 3. The model predicts a higher urine osmolality with active urea secretion than without (1195 vs. 1077 mosm/(kg H
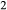
O), respectively). Urine flow rate is predicted to be almost identical in the two situations (86.3 and 85.4 pL/min per nephron, respectively. These flow rates equal 3.28 and 3.25 μL/min per kidney, respectively, assuming 38,000 nephrons per kidney, or 9.45 and 9.36 mL/day per rat. These values are consistent with those reported in rat studies in vivo (Pennell et al. 1974; Bouby et al. 1996). Urea itself contributes to 35% of overall urine osmoles in the base case and 50% with active urea secretion, which is in good agreement with in vivo animal data (see Table 2 in Bouby et al. 1996).

### Case without urea secretion

In the base case, tubular fluid flow and concentration profiles are similar to the base-case profiles described in Layton et al. (2012). In most structures, the model shows a progressive 2.5-fold rise in osmolality along the OM and a 1.5-fold rise along the IM. It is noteworthy that this case yielded a urine osmolality lower than the model in Layton et al. (2012) (1155 mosm/(kg H
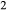
O)), which assumed the same tubular and vascular transport parameters, but did not represent RBCs explicitly. Instead, in Layton et al. (2012) the RBCs were assumed to be infinitely permeable to water and solutes, and their contents were merged with plasma. In the present base case, RBCs were assumed to be highly permeable to urea and water, but impermeable to Na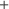. Because these permeabilities are finite, solute concentrations of RBCs lagged those of the surrounding plasma. When RBC permeabilities were assumed to be infinitely large, as in Layton et al. (2012), the lags in RBC solute concentrations were eliminated. As a result, the dissipative effects of the vascular countercurrent exchange on the medullary gradient were reduced, and thereby the concentrating capability of the model was improved.

**Table 3:** A comparison of model predictions without and with active urea secretion. Differences in model predictions are given as percentage of values computed without active urea secretion

Urine	Without urea secretion	With urea secretion	Difference (%)
Urine composition			
Osmolality (mosm/[kg H_2_O])	1077	1195	+11.0
U_urea_ (mmol/L)	388	591	+52.3
U_Na_ (mmol/L)	298	251	−15.8
U_NR_ (mmol/L)	82.3	87.2	−6.0
Excretion rates			
Flow rate (nL/[min · nephron])	0.0854	0.0863	+1.1
Osmole (pmol/[min · nephron])	92	103	+12.0
Urea (pmol/[min · nephron])	33	51	+54.5
Na 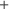 (pmol/[min · nephron])	25	22	−11.9
NR (pmol/[min · nephron])	7.03	7.53	+7.11

A substantial radial osmolality gradient was predicted in the inner stripe, with interstitial fluid osmolality in the interbundle regions higher than in the vascular bundles at the same medullary depth. That radial separation is due to the vigorous active NaCl reabsorption of the thick ascending limbs, which lie distant from the vascular bundles. Osmolality differences are much smaller among the three IM regions, even in the upper IM where tubules and vessels still exhibit clearly identifiable organization, because the thin ascending limbs have no significant active NaCl reabsorption. Owing to the high DVR water and solute permeabilities and to the large AVR fenestration fraction, vascular fluid osmolality closely follows the osmolality and concentrations of local interstitial fluid.

### Case with urea secretion

When active urea secretion is added to the pars recta, urea flow in the long descending limb (LDL) rises from 44 pmol/min per nephron at the cortico-medullary border to 93 pmol/min at the outer–inner stripe border, and urea flow in the short descending limb (SDL) from 73 to 109 pmol/min (see Fig. 2C). Taking into account water reabsorption along those segments, urea concentrations in LDL and SDL rise by 2.55 and 2.06 times, respectively, along the outer stripe (Fig. 1A1).

UT-A2 was found to be expressed along the lower half of the inner stripe segment of the SDL (Wade et al. 2000); thus, we assume that sub-segment to be highly urea permeable (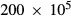 cm/sec). Along the initial approximately 2/3 of this UT-A2-positive subsegment of the SDL, urea is reabsorbed; however, closer to the OM-IM border, where AVR bring in urea-rich fluid from the IM, interstitial urea concentration exceeds that of SDL fluid, and urea is secreted into the late SDL instead (Fig. 2C). Overall, the result of these opposite movements predicted by the model is a net urea reabsorption rate of 6.17 pmol/min per nephron from the whole SDL. In contrast, owing to the increasing interstitial axial gradient of urea concentration in the IM, substantial urea entry is predicted along the initial IM LDL segment, up to 179 pmol/min for the longest LDL. Urea secretion continues along the remainder of the IM LDL segment, which is assumed to be highly urea permeable and which encounters an axially increasing interstitial urea concentration gradient.

Compared to the base case, the model predicted a 11% rise in urine osmolality when active urea secretion was added, but only minimal change in urine flow rate (see Table 3). Interestingly, a marked increase in urea excretion rate was observed (from 33 to 51 pmol/min per nephron) along with a modest reduction in the excretion rate of Na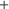 and a modest rise in that of NR. The increase in urine urea concentration, from 388 to 591 mmol/L, resulted from an increased delivery of urea into the IM, via the LDLs. The urine concentrations of Na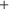 and NR were only modestly affected (Table 3).

With active urea secretion, LDL urea flow at the OM-IM border is predicted to be 119% higher than in the case without active urea secretion (93 vs. 43 pmol/min, respectively). Urea flow at the SDL loop bend with urea secretion (150 pmol/min per nephron) is 45% higher than without urea secretion. As a result, 43% more urea is delivered into the cortical thick ascending limb of short loops with active urea secretion than without. The difference in urea delivery is even more dramatic in the ascending limbs of long loops: 195% more with active urea secretion than without. The impact of urea secretion is more pronounced along long loops because, like they are in vivo, the simulated juxtamedullary nephrons in the model are assumed to be more tortuous and thus longer, providing a larger surface area for transport.

**Figure 3:**
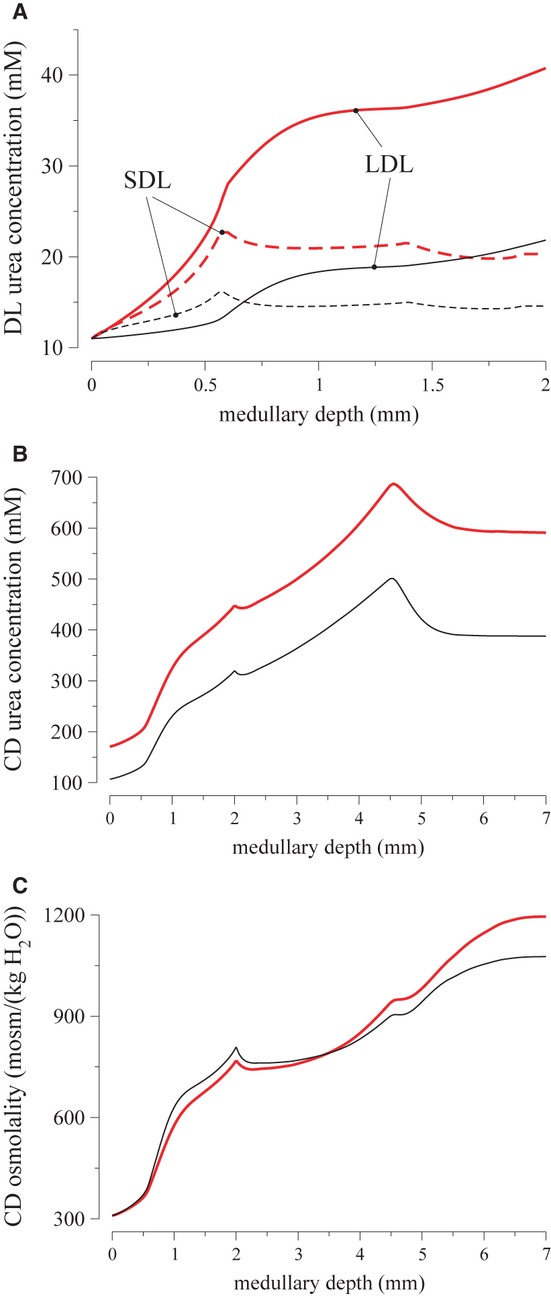
Effects of active urea secretion into par recta. Descending limb and CD fluid concentrations, with active urea secretion (thick red lines) and without (thin black lines). A, short and long descending limb urea concentrations (dashed and solid lines), respectively; B, CD urea concentration; C, CD fluid osmolality. Active urea secretion raises descending limb and CD urea concentrations, as well as the model's overall concentrating capability.

The model assumes that 28% of the urea delivered to the early distal tubule by the cortical ascending limb is reabsorbed in the cortex. As a result, OMCD urea flow rate is substantially higher with active urea secretion than without; and because only a relatively small amount urea is reabsorbed from the OMCD, twice as much urea is delivered to the IM via the CD in the case with urea secretion than in the base case. A comparison of descending limb and CD urea concentration profiles for the two cases can be found in Figure 3A and B.

Note that the urea that flows in AVRs has two different origins: (i) urea delivered to the medullary circulation by the efferent arterioles of the juxtamedullary glomeruli (in plasma that has not been filtered) branching into DVRs, and (ii) previously filtered urea delivered to the IM by the terminal IMCD. Thus, active secretion in the pars recta of urea extracted from the AVRs, can take place even in the absence of previous urea accumulation in the IM, or can exceed the urea available through recycling of previously filtered urea.

**Figure 4:**
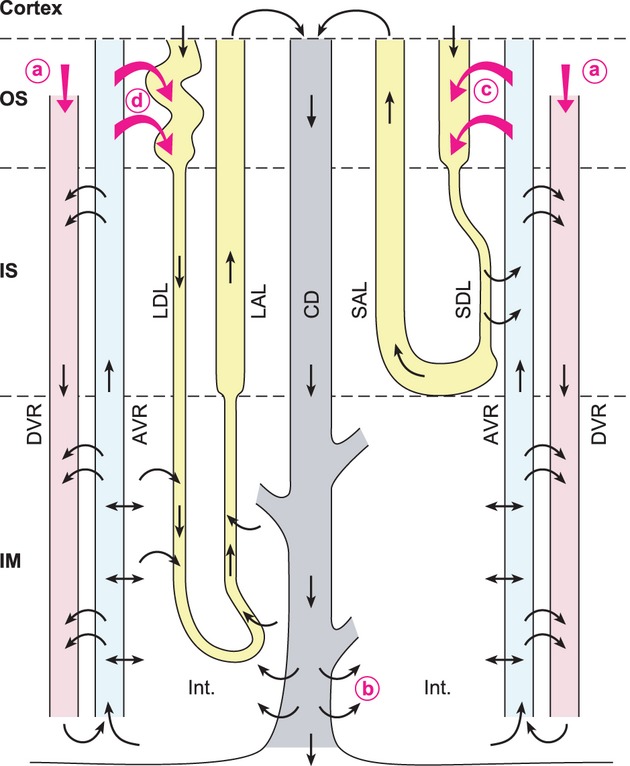
Schematic representation of the fate of urea secreted into the pars recta. A short-looped and a long-looped nephron are represented, as well as two descending and ascending vasa recta (DVR and AVR, respectively). Not shown here: the DVRs in the outer stripe (OS) are main branches of the efferent arterioles of the deep glomeruli and have only limited surface area for contact with the AVR. In contrast, in the inner stripe (IS), Numerous DVRs and AVRs are closely packed in vascular bundles, a configuration that increases contact area and favors countercurrent exchanges. DVRs bring into the medulla plasma that has not been filtered and that contains urea (a). Note that DVRs express UT-B and AVRs are fenestrated. This allows very rapid and efficient exchanges between both structures. In rodents, the short descending limbs (SDL) in the IS are close to the vascular bundles (rat) or are even incorporated among the AVRs and DVRs, forming so-called “complex vascular bundles”. IM segments of long loops are assumed to have high urea permeabilities, consistent with preliminary data in rat (Pannabecker and Dantzler, pers. comm.). The present model assumes that urea is actively secreted in the pars recta of both short-looped (c) and long-looped (d) nephrons. Some of this urea can be added to the urea that cycles in the renal medulla, brought to the interstitium via the terminal IMCD) (b). This improves the ability to accumulate urea in the deep IM and to selectively concentrate urea in the urine. Abbreviations: LDL/LAL, long descending/ascending limb; SAL, short ascending limb; CD, collecting duct; IM, inner medulla; Int. interstitium.

Figure 4 describes the fate of the urea secreted into the pars recta of a short-looped and a long-looped nephron. The secreted urea is brought to the IM via different routes. It runs along the nephron (of both short and long loops) until it reaches the CD and is delivered to the IM. In addition, in the short loop, some of the urea flowing in the SDL diffuses in the AVR and is then submitted to countercurrent exchange with DVR which bring this urea down to the IM. Because UT-A2 is expressed only in the lower half of the SDL, this countercurrent exchange can occur in the vascular bundles of the upper half of the IS, preventing its return toward the venous renal blood. Thus, urea secretion allows an additional delivery of urea to the IM via both a tubular and a vascular route.

It is noteworthy that active urea secretion enhances the concentrating mechanism, as measured by an increase in CD tubular fluid osmolality in the IM, but not in the OM. Indeed, OMCD fluid osmolality rises slightly more in the base case than in the case with active urea secretion; see Figure 3C. In contrast, tubular fluid osmolality along the IMCD increases by a factor of 1.55 in the case with urea secretion and only by a factor of 1.33 without this secretion. Altogether, the results of this simulation suggest that active urea secretion improves urine concentrating ability by amplifying urea cycling within the medulla, and allows the kidney to excrete a higher load of urea without requiring additional water and with only a minor reduction in the excretion of other solutes.

**Figure 5:**
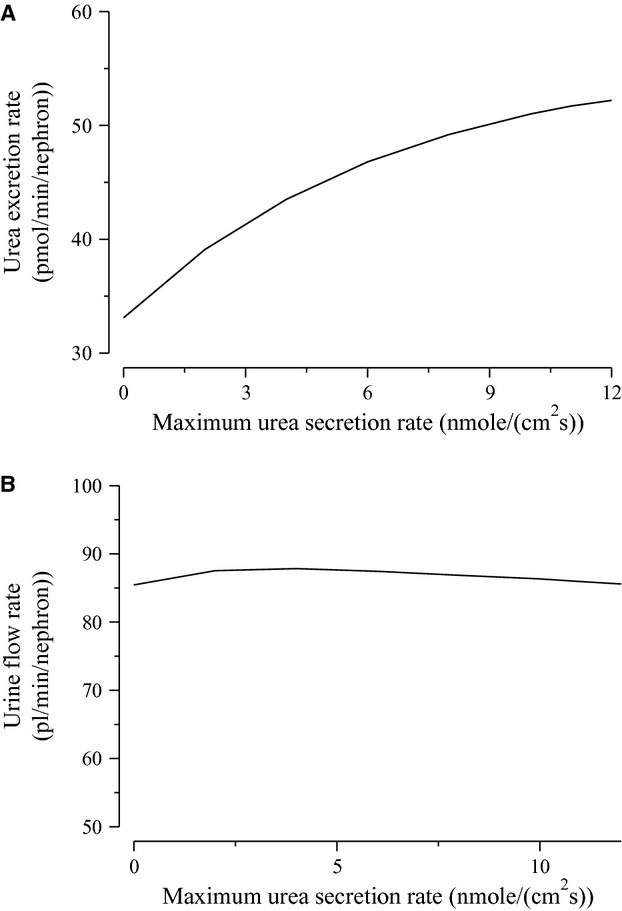
Urinary flow rate (A) and urea excretion rate (B) as functions of maximum active urea secretion into pars recta (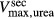). Results suggest that active urea secretion selectively increases urea excretion rate without increasing urine flow.

To further demonstrate the impact of active urea secretion on urea and fluid excretion rates, we computed model solution for different values of maximum active urea secretion rate, 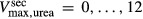 nmol/(cm
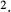
sec). (Recall that the baseline value for 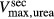 was set to 10 nmol/(cm
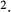
sec) in the “With urea secretion” case.) As expected, the model predicted a rise in urea excretion rate as active urea secretion rate increases; see Figure 5A. As the 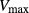 of urea secretion increases from 0 to 12 nmol/(cm
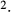
sec), urea excretion rate increases by 1.7-fold, from 33.1 to 52.2 pmol/(min · nephron). In contrast, for the whole range of 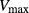 considered, urine flow rate varies by less than 3%, and exhibits a non monotonic pattern (slightly decreasing with increasing 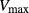 in the upper range of 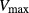) (Fig. 5B).

## Discussion

We have extended a mathematical model of the urine concentrating mechanism in the rat kidney to evaluate the impacts of a hypothesized active urea secretion in the pars recta of the proximal tubule. In this model, transmural transport by tubules and vessels is approximated by single-barrier expressions that summarize experimental results for osmotically driven water fluxes, solute diffusion, and active solute transport. The model, which was solved to steady state, predicts, in all represented structures, concentrations of the solutes represented, the osmolality arising from those solutes, the intratubular (or intravascular) flow rates of water and solutes, the transmural fluxes of water and solutes, and the excretion rates of solutes. The main findings revealed by this simulation is that an active urea secretion in the pars recta allows the kidney to excrete a greater amount of urea without increasing the amount of water required for this excretion and with only a small influence on the excretion of other solutes. This “urea selective” improvement in the kidney's concentrating ability renders the model results very similar to in vivo observations.

The conclusion that active urea secretion may introduce “urea selective” improvement in the kidney's concentrating ability was drawn based on the model's prediction of a 55% increase in urinary urea excretion, together with a modest decrease in salt excretion, with proximal tubular active urea secretion. An alternative approach to assess the impact of active era secretion is to match the solute excretions in the two cases, and then compare their urine osmolalities. However, to match the urinary excretion rates would require using different model parameters or boundary conditions in the two cases, which we feel would render it difficult to conclude whether any difference in model urine osmolality is due to active urea secretion or the different parameter values.

The existence of an active urea secretion into the proximal straight tubule of some mammalian kidneys is suggested by a number of functional observations in mice, rats, dogs, and humans (Bankir and Trinh-Trang-Tan 2000; Bankir and Yang 2012). However, direct experimental evidence for active urea secretion has been shown in only one study of isolated perfused pars recta of the rabbit kidney, and this secretion was quantitatively small (Kawamura and Koko [Bibr b14]). Another study, also in the rabbit kidney, did not confirm this result (Knepper 1983). Importantly, the first study dealt with medullary and cortical pars recta while the second study used only cortical pars recta. These two subsegments are not exposed to the same vascular beds in situ and, as explained in Bankir and Yang (2012), the outer medullary environment is more favorable to a possible secretion. At the time of these experimental studies, rabbit was the only species in which isolated perfused tubule experiments were feasible. But, as a herbivore eating a protein-poor diet, the rabbit has a greater need for nitrogen conservation than for efficient nitrogen excretion. Indeed, in herbivores (Schmidt-Nielsen et al. 1958), as well as in rats fed a low protein diet for several weeks (Isozaki et al. 1994; Sands et al. 1996), active urea reabsorption has been observed to take place along the upper IMCD. In carnivores and omnivores, active urea secretion instead likely occurs in another segment of the nephron to improve nitrogen excretion. In support of this view, many studies reported fractional excretion of urea exceeding 50% in rats, dogs, and humans (see table 2 in Bankir and Yang 2012) and two studies reported fractional excretion of urea exceeding 100% in mice (Fenton et al. 2005; Yang and Bankir 2005).

To our knowledge, there has been no attempt yet to characterize a possible urea secretion by in vitro microperfusion of pars recta isolated from the kidney of rats, mice or any other omnivore or carnivore. In the absence of such experimental data, this study, which simulates an active urea secretion in the rat kidney, offers an opportunity to characterize the consequences of this secretion. Addition of this secretion to our previous model of the rat kidney medulla (Layton et al. 2012) resulted in a marked increase in urine urea concentration, a modest fall in the concentration of sodium and a modest increase in urine osmolality with almost unchanged urine flow rate. As a result, the excretion rate of urea was much higher in the presence of urea secretion, while the excretion rate of other solutes was only modestly altered.

Model results are in good agreement with in vivo data obtained in a study by Safirstein et al. (1981). Administration of cisplatin, a drug that accumulates in pars recta cells, completely blocked the addition of urea in the loop of Henle. In cisplatin-treated rats, a net urea reabsorption was observed between the late proximal tubule and the early distal tubule accessible to micropuncture at the kidney surface, instead of a net urea addition observed in normal rats. A substantial selective reduction in urea excretion rate and a rise in sodium excretion rate were observed in these experiments (Safirstein et al. 1981), similar to the effects revealed by the model when comparing the base case with the case with active urea secretion. Several studies suggest that active urea secretion also occurs in humans. One such example is “familial azotemia,” in which affected members exhibit an isolated threefold increase in serum urea concentration, a markedly reduced fractional excretion of urea, but a normal creatininemia and no sign of kidney dysfunction. One explanation may be a loss-of-function mutation in the membrane transporter responsible for active urea secretion (Hays 1978; Bankir and Yang 2012).

If translated to a living rat, the marked increase in urea excretion rate seen in our model with active urea secretion would deplete the body stores of urea and induce a fall in plasma urea concentration so that the resulting decline in filtered urea would bring the whole system to a new steady state with a higher fractional excretion of urea. The case without urea secretion, compared to the case with active urea secretion, reproduces the phenotype seen in humans with familial azotemia, including an elevated uremia, a less efficient urea excretion, and a modest reduction in urine concentrating ability.

For a long time, it was assumed that some of the urea ascending from the IM in the AVR was “recycled” (re-introduced) in the thin limbs of short loops of Henle that express UT-A2 and that run in close proximity of the vascular bundles in rats, mice and desert-adapted rodents (Kriz 1981; Bankir and de Rouffignac 1985). This urea addition was thought to account for the higher urea flow measured in the early distal tubule than in the late proximal tubule accessible to micropuncture (De Rouffignac and Morel 1969; Armsen and Reinhardt 1971; de Rouffignac et al. 1981; Bankir and Yang 2012). However, the phenotypes described recently in UT-A2 knockout mice (Uchida et al. 2005) and in double UT-A2/UT-B knockout mice (Lei et al. 2011) are not compatible with these assumptions. According to the previous concept, abolition of UT-A2 permeability should have induced a significant urine concentrating defect that UT-A2 knockout mice did not exhibit. Moreover, contrary to what could be expected from the classical “urea recycling” concept, the abolition of UT-A2 permeability in the double knockout mice, instead of aggravating further the urine concentrating defect, restored a nearly normal urine concentrating ability, which is severely impaired in the simple UT-B knockout mice (Lei et al. 2011). The present model simulations suggest that a net urea reabsorption occurs in the SDL when active urea secretion into the pars recta increases the flow of urea delivered to the thin limbs.

It has been proposed that the role of the SDL UT-A2 may lie in the transient buildup of a urea and osmolality gradient in the IM, rather than in the generation of the steady-state gradients (Lei et al. 2011; Bankir and Yang 2012). That is because for the urea in SDL to return to the IM, the transit time required for UT-A2-mediated reabsorption and the course through the DVR is shorter than the transit time through the thick ascending limb and CD.

Also, in the above transient state, transepithelial urea flux through the long loop epithelium may occur in an opposite direction from that in an established antidiuretic state considered in the present model. The IM segments of the model loops are assumed to have high urea permeabilities, consistent with preliminary data in rat (Pannabecker and Dantzler, pers. comm.). In water diuresis, an almost normal sodium chloride gradient is present in the IM, but the urea gradient is absent (Saikia 1965; Valtin 1966). When vasopressin action is restored, the urea gradient builds up slowly by delivery of concentrated urea through the vasopressin-dependent urea transporters of the terminal collecting duct. When the urea gradient is being built up in the IM, active urea secretion into the proximal tubule of the long loops may yield a transepithelial gradient favorable for urea delivery by the LDL into the interstitium (opposite to the direction shown in Fig. 4), thus adding to the urea delivered to the IM insterstium through the terminal IMCD.

Owing to urea's mild toxicity (Coombe et al. 1960; Word et al. 1969; Bankir and Trinh-Trang-Tan 2000; Bankir and Yang 2012), evolution might have favored a low plasma urea concentration in mammals. This presents a challenge for the kidney of a non-herbivorous mammal to raise urea concentration in urine far above that in plasma. Active urea secretion may be an adaptation that allows the kidney to face this challenge, as opposed to the active urea reabsorption that allows the reuse of urea nitrogen in species with a low protein intake. Taken together, the results of the present simulations suggest that active urea secretion selectively improves the ability to concentrate urea in the urine, thereby allowing a greater amount of urea to be excreted without a change in urine flow rate, that is without a greater water requirement. This secretion results in a “urea-selective” improvement in urine concentrating ability.

In summary, this study, using an elaborate model of the rat renal medulla, strongly suggests that an active urea secretion in the pars recta of the proximal tubule takes place in the rat kidney and that it allows a more efficient excretion of urea with negligible impact on the amount of water required for this excretion. In vivo micropuncture studies in rats and mice, as well as microperfusion experiments of isolated medullary pars recta in vitro are required to confirm these modeling results. We hope these “in silico” results will stimulate further experimental research.
